# Letter to the Editor regarding the article ‘ER stress-related protein, CHOP, may serve as a biomarker of mechanical asphyxia: a primary study’ by Hu and colleagues

**DOI:** 10.1007/s00414-022-02808-y

**Published:** 2022-03-01

**Authors:** Alberto Chighine, Emanuela Locci, Giulio Ferino, Fabio De-Giorgio, Ernesto d’Aloja

**Affiliations:** 1 Department of Medical Sciences and Public Health, Section of Legal Medicine, University of Cagliari, Cagliari, Italy; 2 Department of Health Surveillance and Bioethics, Section of Legal Medicine, Catholic University of Rome, Rome, Italy; 3 Fondazione Policlinico Universitario A. Gemelli IRCCS, Rome, Italy

We read with interest the article recently published by Hu and colleagues [1].

Basing on growing evidence linking cellular hypoxia induced by mechanical asphyxia to mitochondrial disfunction, the authors proposed a mitochondria-related protein — namely C/EBP Homologous Protein (CHOP) — as a potential biomarker of asphyxial deaths at very early post mortem intervals.

The tissue-specific expression of this protein — together with the quantification of several end-products of energetic metabolism (adenosine triphosphate, ATP; reactive oxygen products, ROS; mitochondrial membrane potential, MMP) — has been studied in both cell and rodent model, and even in human heart and in brain tissues.

A correlation between the mechanism of death and the expression of CHOP has been highlighted.

These findings objectivize the foreseeable mechanism of damage, and death, during mechanical asphyxia, mainly represented by the irreversible impairment of the cellular energetic powerhouse: mitochondria.

Although on a different perspective, evidence of mitochondrial dysfunction and impairment was already provided by our group while investigating, through a metabolomic approach, a homogeneous animal model of cardiac arrest (CA) [2].

In our design of experiment, one of the two investigated arms reproduces indeed a CA secondary to a pure hypoxic/anoxic insult, obtained by clamping the trachea of landrace pigs.

Despite the experiment was designed, and conducted, with a prevalent clinical interest, the minute-by-minute plasma sampling precisely addresses modifications in the metabolome trajectories of animals experiencing CA secondary to asphyxial mechanism. The results allowed us to distinguish them from those experiencing a CA by ventricular fibrillation.

The identified metabolome modifications suggest a severe impairment of the mitochondrial tricarboxylic acid (TCA) cycle, mainly driven by reverse activity of succinate dehydrogenase (SDH) in a poor oxygen environment, consistently with the best literature available [3].

Of greater forensic interest was the second paper published by our group [4], focused exclusively on the asphyxial phase preceding CA, which describes metabolome modifications according to the animals’ outcome (damage/no damage), providing a comparison with heart and brain histopathology and immunohistochemistry.

Microscopical approach was able to distinguish animals not recovering from CA from those who were still alive — and in good health — at the end of the experiment. Such an approach was useful to identify the severity of the damage, while it was unfitted to identify its causal mechanism, sharing the two models of CA a similar histopathological final pattern of damage.

Plasma metabolome modifications, on the other hand, promptly identified TCA adaptations to mounting hypoxia towards anoxia through changes in metabolomic trajectories, mainly driven by central components of the TCA cycle (namely succinate and malate).

Succinate appears to be the key molecule during oxygen shortage, being accumulated from fumarate reduction, through the reverse activity of SDH/electron transport chain (ETC) complex II, as the final electrons acceptor (in oxygen place itself).

Yet succinate rise embodies early sign of both ATP production necessity (as TCA cycle needs to keep working to generate ATP even in the preliminary phase of oxygen reduction) and radical oxygen species (ROS) overproduction (since, over a certain amount, succinate accumulation cannot be buffered by ETC whenever oxygen levels are restored). Furthermore, MMP changes detected by Hu et al. may be sustained by the well-known mitochondrial permeability transition pore (mPTP) which has been demonstrated to be formed under oxidative stress such as ROS and Ca^2+^ overload secondary to succinate massive increase under hypoxic/ischemic settings [5].

This finding coherently follows the intriguing role played by plasmatic succinate, already described in literature [6], suggesting a dual signalling function according to its concentration: a *pro-survival* role up to certain amount and a *pro-death* one over a point-of-no-return, where the cellular apparatus cannot cope with the upcoming ROS burst.

Furthermore, in our experiment, a significant inter-individual variability in terms of resilience to ongoing hypoxia was highlighted, despite the limited sampling size (*n* = 10).

One of the most intriguing results from a forensic perspective was that asphyxial length before CA did not directly relate to histopathological heart and brain damages nor to poor clinical outcome, while increasing plasmatic hypoxanthine resulted the only metabolite with a time-related trend.

Although driven by succinate and malate plasmatic increase, the metabolomic profile related to asphyxial death was characterised by a wider perturbation of metabolites, which underlines an up- or down-regulation of several metabolomic pathways.

In this perspective, Hu and colleagues’ data strengthen our preliminary results.

Mitochondria end-products represent the final signature of the activation/deactivation of genes, miRNAs, and proteins, and all of them may be implemented as biomarkers in forensic caseworks whenever dealing with asphyxial deaths.

Forensic community should be encouraged to address this paramount issue in forensic pathology by a cooperative attempt to look at this complex biological phenomenon by a different point of view.

A synoptic reading of results from the works of Hu et al. and those from our research group may pave the way to this multidisciplinary approach.
